# Health Ruined

**Published:** 1862-07

**Authors:** 


### HEALTH RUINED.

TWO young men called to consult us to-day at the same time; one from the city, the other from a farm in New-England, both the victims of chronic maladies; the ambition of both, and the hopes of both, blasted and blighted by the inroads of disease; both in their twenty-second year. Whether they can be rescued from a premature grave, time, the great solver of difficult problems, alone can tell. It is hard enough for one to make his own way in the world, even when in vigorous health; but to earn an honest living, and to lay up money for the future, when weighed down by some disease which makes itself felt every hour of waking existence, is in all cases a painful and laborious task; in some, an impossibility. It is natural that the young should be vigorous and strong; but that vigor and that strength are often thrown away by inexcusable ignorance, or unpardonable thoughtlessness. The citizen had come to town with high health, high hopes, and a high ambition, backed by a good family, a good education, and good moral principles. He had a manliness of expression which at once inspired confidence, and secured a profitable situation. Within six months his health was broken, and at the end of three years he is still an invalid, from the following cause—a cause which one would suppose any man of two ideas might have known must inevitably produce pernicious results. His employers occupied a basement in Water street as a counting-room, so damp that it spoiled their goods, which had to be removed on that account: still the place was used for keeping books, writing letters, etc. The young man's duties called him out several times every day, in the heat of summer, never failing to induce free perspiration. As soon as he reached his office, he pulled off his coat and hat, and employed himself at his writing-desk, with the result of cooling off rapidly in a low room known to be damp and cold. He saw at length that his health was declining, and reading some stupid article in a newspaper about the all-efficient benefits of physical exercise, he entered his name in a gymnasium at the beginning of winter. His mode of procedure was as follows: He walked from the store to the gymnasium pretty rapidly, making himself a little warm. Ushered into a cold room, he pulled off his warm clothing, and put on the cold garments supposed to be more suitable for his performance. In an hour or two he was in a delightful glow, a healthful heat, from vigorous exercise; when, getting a little tired, he went to the clothes-room, took off the warm garments, and put on others which were not merely cold, but a little damp from the exercise in coming to the place, and by the time he got out of doors, he was chilled; and this was persisted in until its absurdity was brought to his attention. It is by such thoughtlessness and ignorance that many a young man is sent to an early grave.

The young farmer had a variety of ailments, but, as a saving clause in his estimation, he had a good appetite; and it is not an uncommon thing for patients to complain of aches and pains from top to toe, but to the question, "What's your appetite?" the ready and uniform reply is: "First rate; no fault to find with that." The young farmer said he was weak, couldn't work; in fact, it appeared that all he could do was to grunt and eat, just like a pig. He could eat all day, at regular meals and between times. He always had a good appetite for supper — a New-England supper — the chief elements of which were sodden bread, greasy cakes, pie, preserves, and doughnuts. Reader, be honest; don't you think that such folks ought to die? There is no kind of use for people in this world who have no brains. More than twenty years ago we made the tour of New-England, just to be seeing things; and such dishes of fat pork and fries, sweet doughnuts boiled in hog's fat, sour preserves, (that is, sweetened fruits in a state of decay,) and bread weighing an ounce to the cubic inch, we never saw before nor since, and never want to. Is New-England intelligence on a wool-gathering expedition still, that they have "pie" for supper? What is a "pie," as generally made? Even after all the cooking it gets, it is stewed fruit, covering over a layer of mere dough, almost as heavy as lead, almost as indigestible as a piece of raw-hide or India-rubber. Two simple rules in relation to this subject would prevent an incalculable amount of pain and suffering: First. Eat nothing between meals. Second. Take no supper at all, or if any thing, only a single cup of weak tea and crust of cold bread and butter.

